# Supplementary material for: Selective BET bromodomain inhibition as an antifungal therapeutic strategy
Source: Nat Commun. 2017 May 18;8:15482. doi: 10.1038/ncomms15482 (PMC5454392; doi:10.1038/ncomms15482)
Supplement: Supplementary Information — Supplementary Figures, Supplementary Tables, Supplementary Methods and Supplementary References [file ncomms15482-s1.pdf]

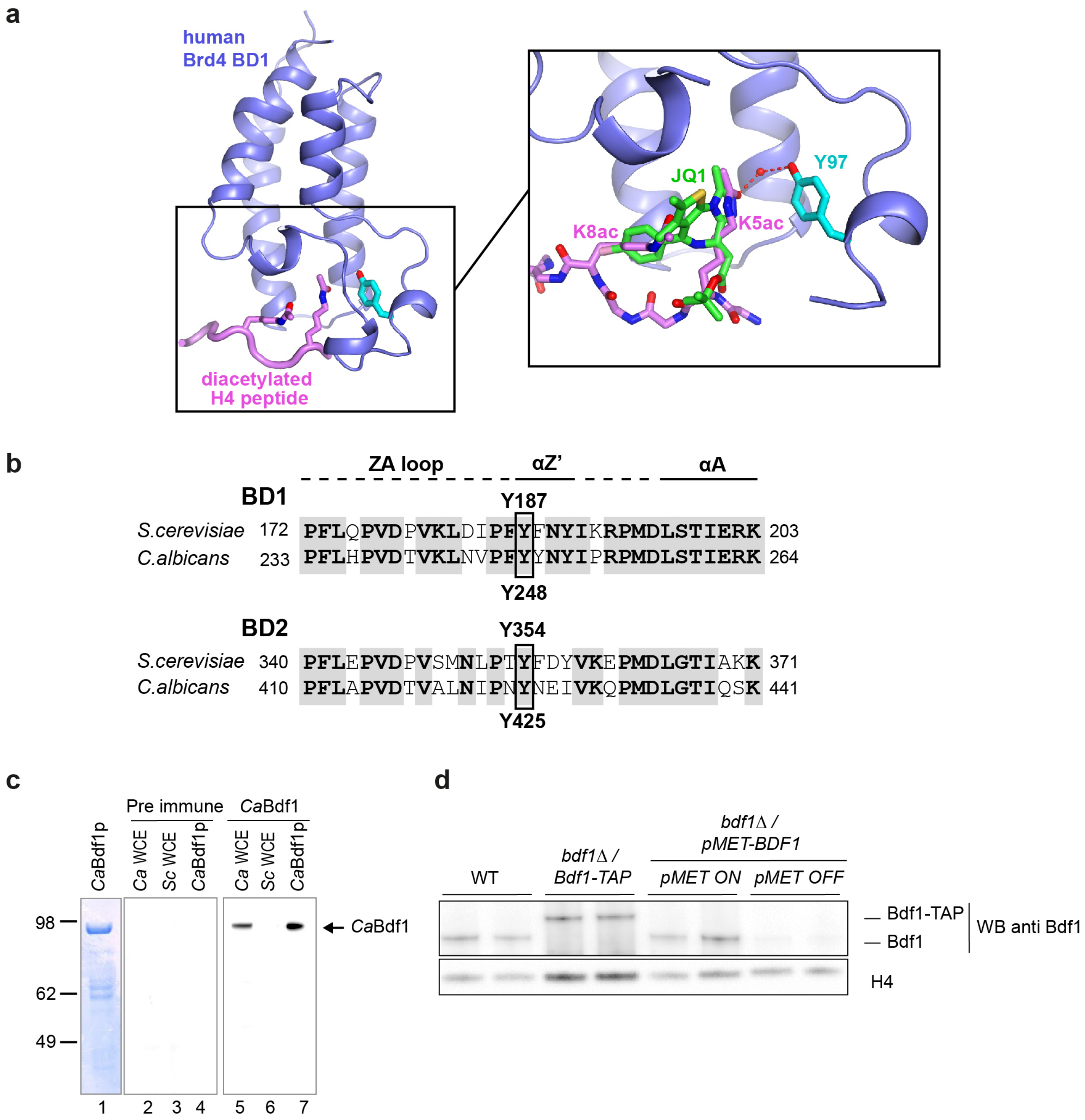

Supplementary Figure 1. BET BD structure, YF mutations and development of an anti-*Ca*Bdf1 antibody. (**a**) Structure of human Brd4 BD1 bound to an H4 peptide diacetylated on lysines 5 and 8 (PDB ID 3UVW)[1]. The closeup view shows the superposition with Brd4-BD1 bound to JQ1 (PDB ID 3MXF)[2]. Tyr97 forms a water-mediated hydrogen bond with the acetyl group of Lys5 and is critical for peptide ligand recognition. (**b**) Location of the YF point mutations used in this study. The mutated Tyr residues correspond to residue Tyr97 in Brd4-BD1. (**c**) Development of an antibody specific to *C. albicans* Bdf1. A band of the expected molecular weight is specifically detected after immunization of the rabbit. WCE, whole cell extracts. (**d**) Validation of antibody specificity. The antibody detected a shifted band when Bdf1 was TAP-tagged, whereas the signal was lost when Bdf1 expression was inhibited. The full blots are shown in **Supplementary Fig. 13c**.

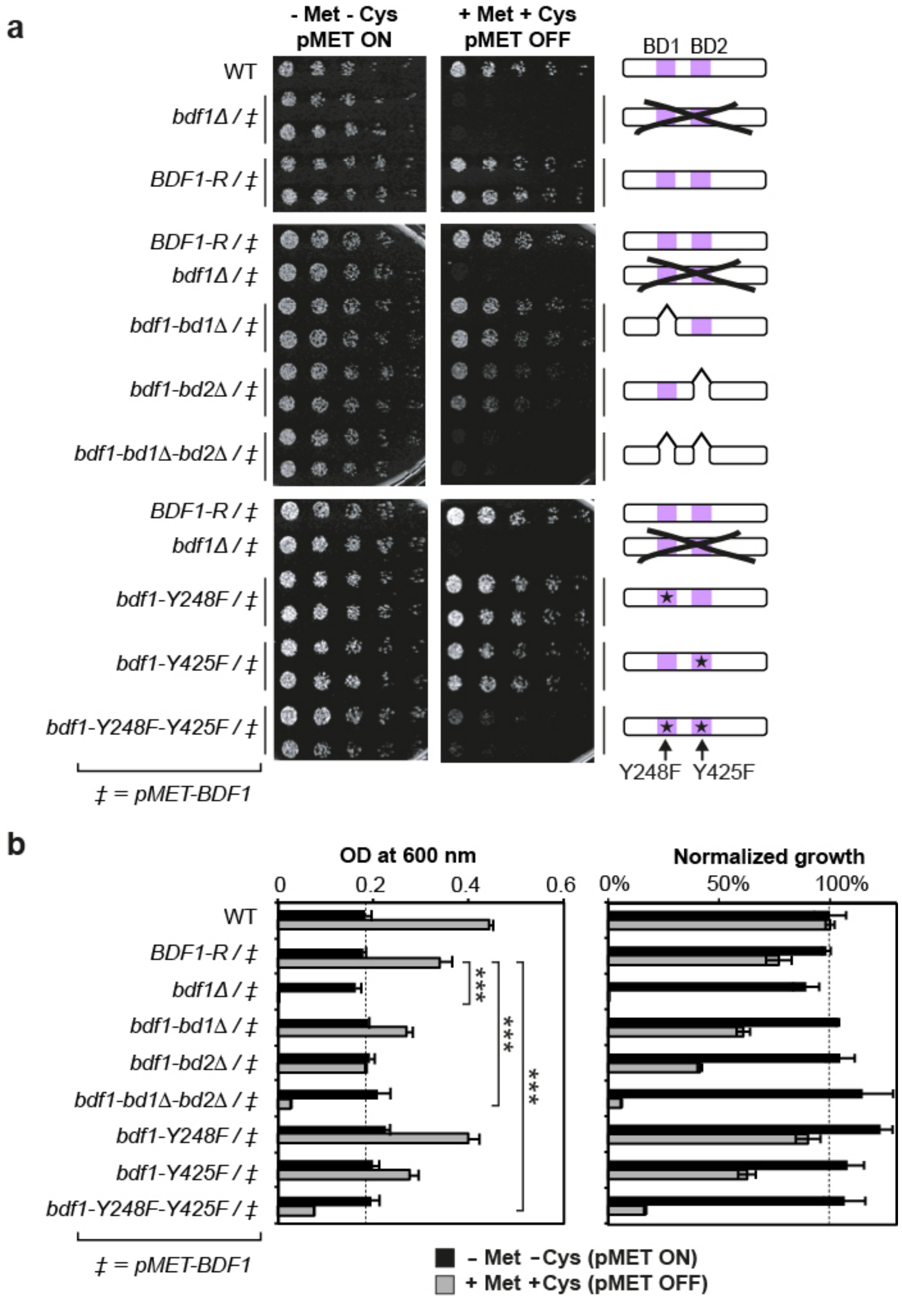

Supplementary Figure 2. Bdf1 BDs are essential in *C. albicans*. (**a**). Colony formation assay of strains in which Bdf1 expression is controlled by a methionine-sensitive promoter (*pMET*). The viability of *C. albicans* is lost when Bdf1 is absent or when both Bdf1 BDs are mutated. Met, methionine; Cys, cysteine. (**b**) Liquid growth assay using the strains shown in (a). *Left*, unnormalized OD. The repression of the *pMET* promoter requires the addition of high concentrations of methionine and cysteine, which affects the growth of *C. albicans* (WT, compare black and grey bars). Data represent the mean and s.d. values from three independent experiments. ***, $P \leq 0.01$. *P*-values were determined using a two-sided Welch *t*-test. *Right*, data were normalized to the WT growth (both black and grey bars are equal to 100% in the WT strain).

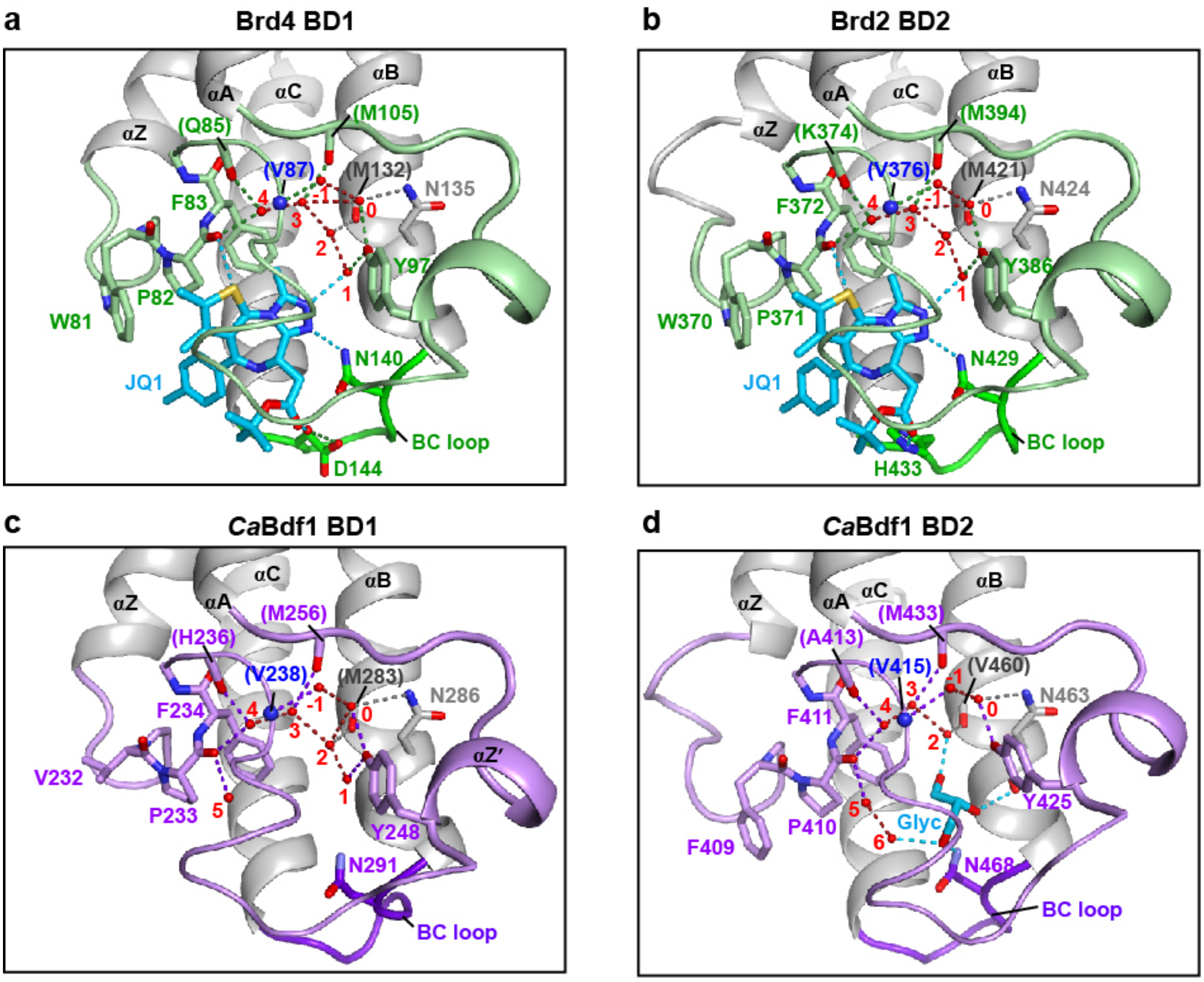

Supplementary Figure 3. **Conserved water molecules in human and *C. albicans* BET BD active sites.** Structures are shown for (**a**) human Brd4 BD1 bound to JQ1 (PDB code 3MXF), (**b**) human Brd2 BD2 bound to JQ1 (PDB code 3ONI), (**c**) *C. albicans* Bdf1 BD1 and (**d**) *C. albicans* Bdf1 BD2. ZA and BC loops are colored green (a,b) or violet (c,d)**.** JQ1 bound to the human BDs and a glycerol (Glyc) molecule bound to *Ca*Bdf1 BD2 are shown in cyan. Conserved water molecules are numbered as in ref.[3]. Water molecule 1 is missing from *Ca*Bdf1 BD2, presumably dislodged by the bound glycerol molecule. The invariant Asn and Tyr residues, hydrophobic shelf and residues interacting with water molecules are shown in stick representation. Backbone atoms are labelled in parentheses. Hydrogen bonds to bound ligands are in cyan; those from water molecules to an amino acid residue are in the colour of the residue; and those between water molecules are in red.

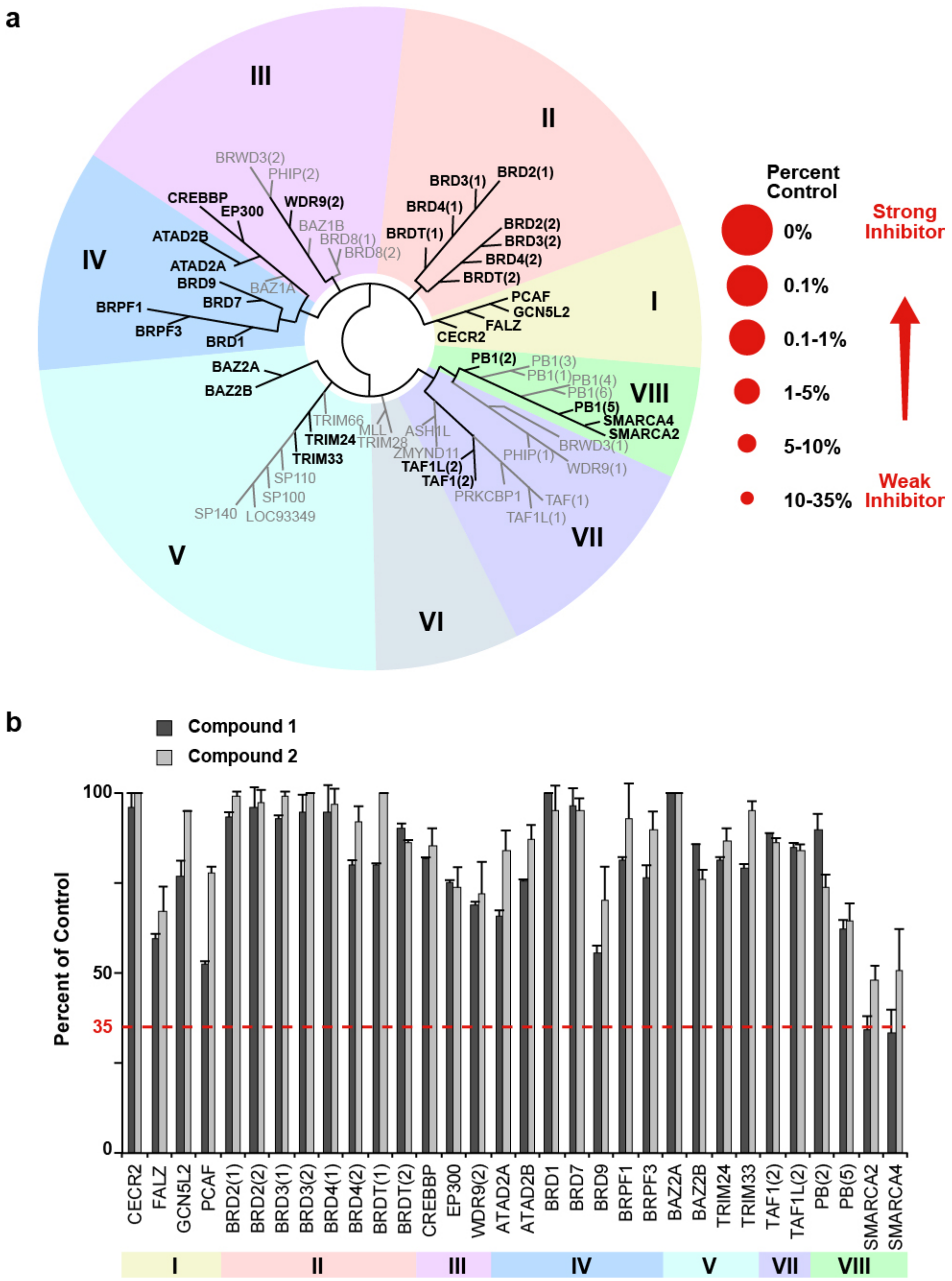

Supplementary Figure 4. BROMOscan profiling of compounds 1 and 2 at 10 μM concentration. (**a**) TREEspot interaction map. Roman numerals indicate human BD families as defined in ref.[1]. The 32 BDs used in the screen are shown in black. Results of the BROMOscan are plotted as percent of control, where lower values indicate stronger inhibition. No spots appear on the plot because no significant inhibition was detected. (Plots are identical for compounds **1** and **2**). For comparison, the BET-selective inhibitor dBET1 (a derivative of JQ1), was reported to yield signals of 0% for six BET BDs (0.25% and 1.6% for the other two), 25-35% for CREBBP, EP300, FALZ and SMARCA4, and >35% for the remaining BDs[4]. (**b**) Histogram of BROMOscan data. Results show mean and s.d. from two independent experiments. The red dashed line indicates the 35% threshold used as a cutoff for significant inhibition.

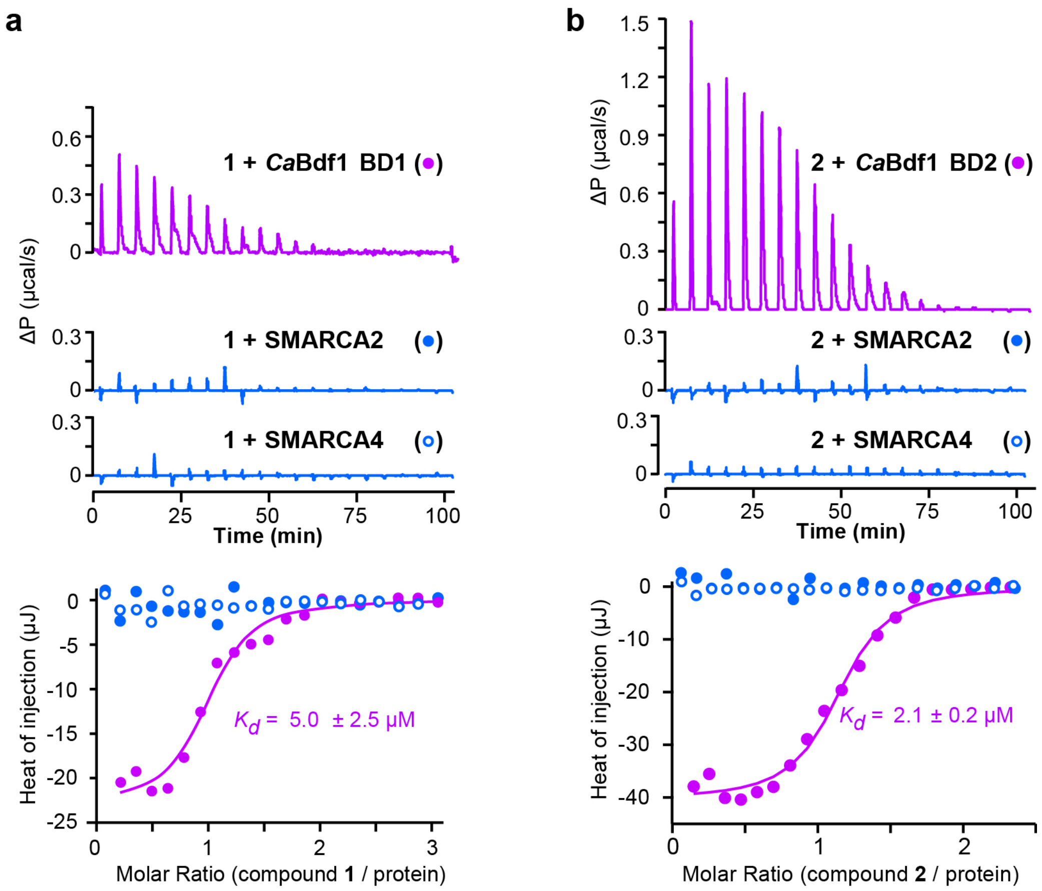

Supplementary Figure 5. ITC experiments measuring the binding of compounds 1 and 2 to human SMARCA BDs. The binding of (**a**) compound **1** by *Ca*Bdf1 BD1 and of (**b**) compound **2** by *Ca*Bdf1 BD2 is compared to the binding of these compounds by SMARCA2 and SMARCA4. The upper panels show the differential power (ΔP) time course of raw injection heats for *Ca*Bdf1 (magenta) and SMARCA (blue) BDs. The lower panels show normalized binding enthalpies corrected for the heat of dilution as a function of binding site saturation, with *Ca*Bdf1, SMARCA2 and SMARCA4 BDs indicated by magenta, solid blue and empty blue circles, respectively. The ITC data for the *Ca*Bdf1 BD1 and BD2 are those of **Fig. 6d** and **7c**, respectively.

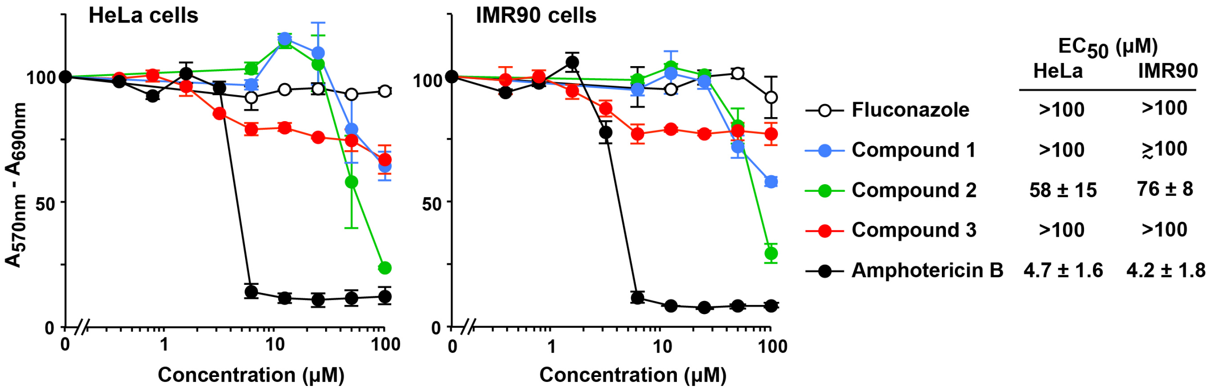

Supplementary Figure 6. MTT cytotoxicity assays on HeLa and IMR90 (primary fibroblast) cells. Antifungal compounds fluconazole and amphotericin B were included as controls. Data represent the mean and s.d. values from three independent experiments. The values of median effective concentration (EC_{50}) are summarized on the right.

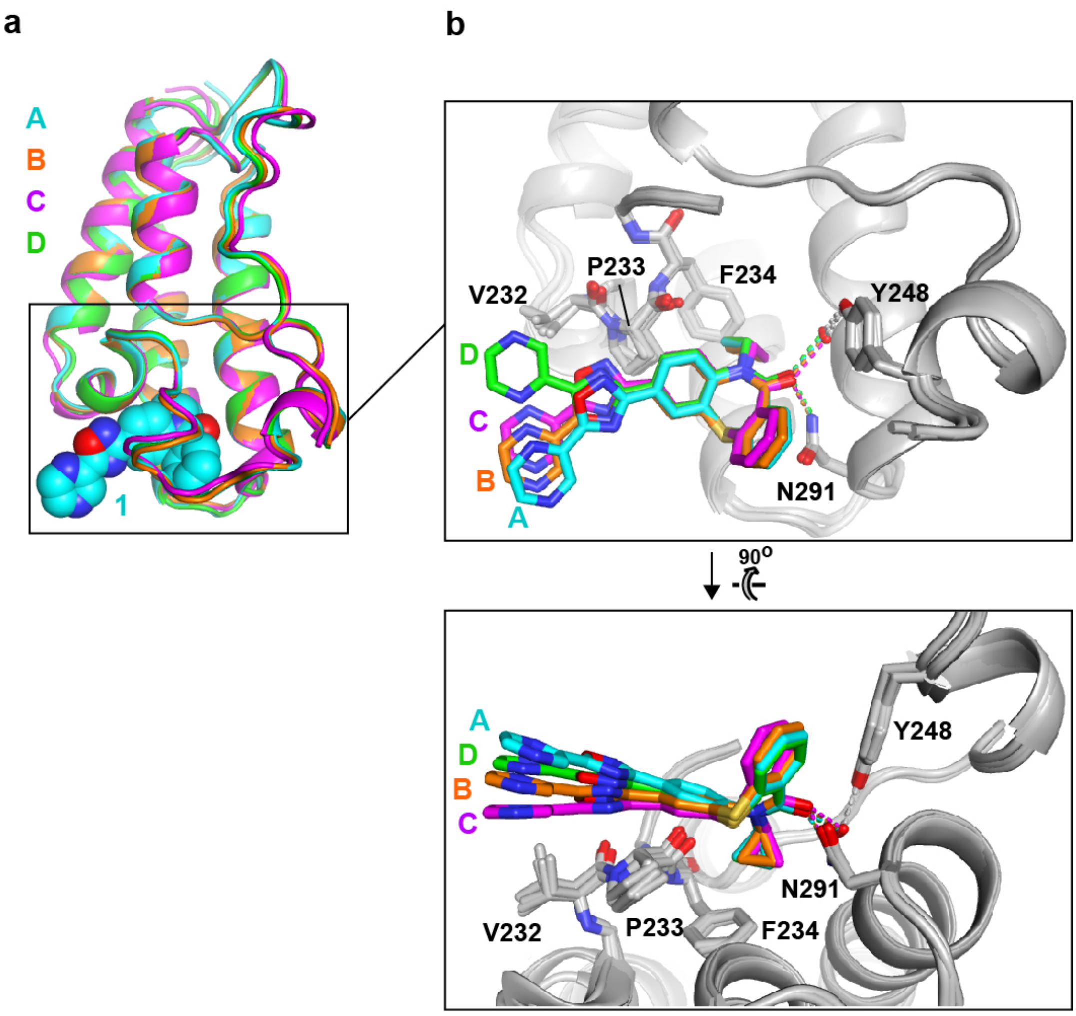

Supplementary Figure 7. Conformations of compound 1 observed in the *Ca*Bdf1 BD1 crystal structure. (a) Structural alignment of the four *Ca*Bdf1 BD1 polypeptide chains present in the asymmetric unit, each bound to a molecule of **1**. Chains A, B, C and D are coloured cyan, orange, magenta and green, respectively. (**b**) Orthogonal views of the binding site showing the different conformations of the bound ligand.

a

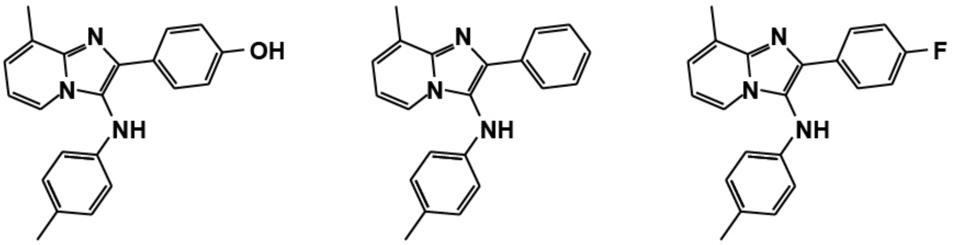

2 2a 2b

b

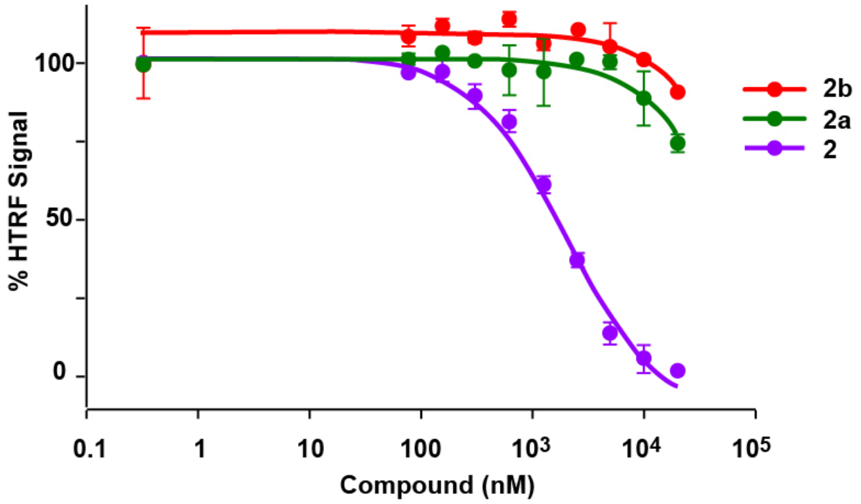

Supplementary Figure 8. Importance of the hydroxyl group in 2 **for inhibition of *CaBdf1* BD2.** (**a**) Chemical structure of compound **2** and analogs in which the hydroxyl group is replaced by hydrogen or fluorine. (**b**) HTRF assays showing the effect of analogs of **2** on the acetylpeptide binding activity of *Ca*Bdf1 BD2. Data represent the mean and s.d. from three independent experiments.

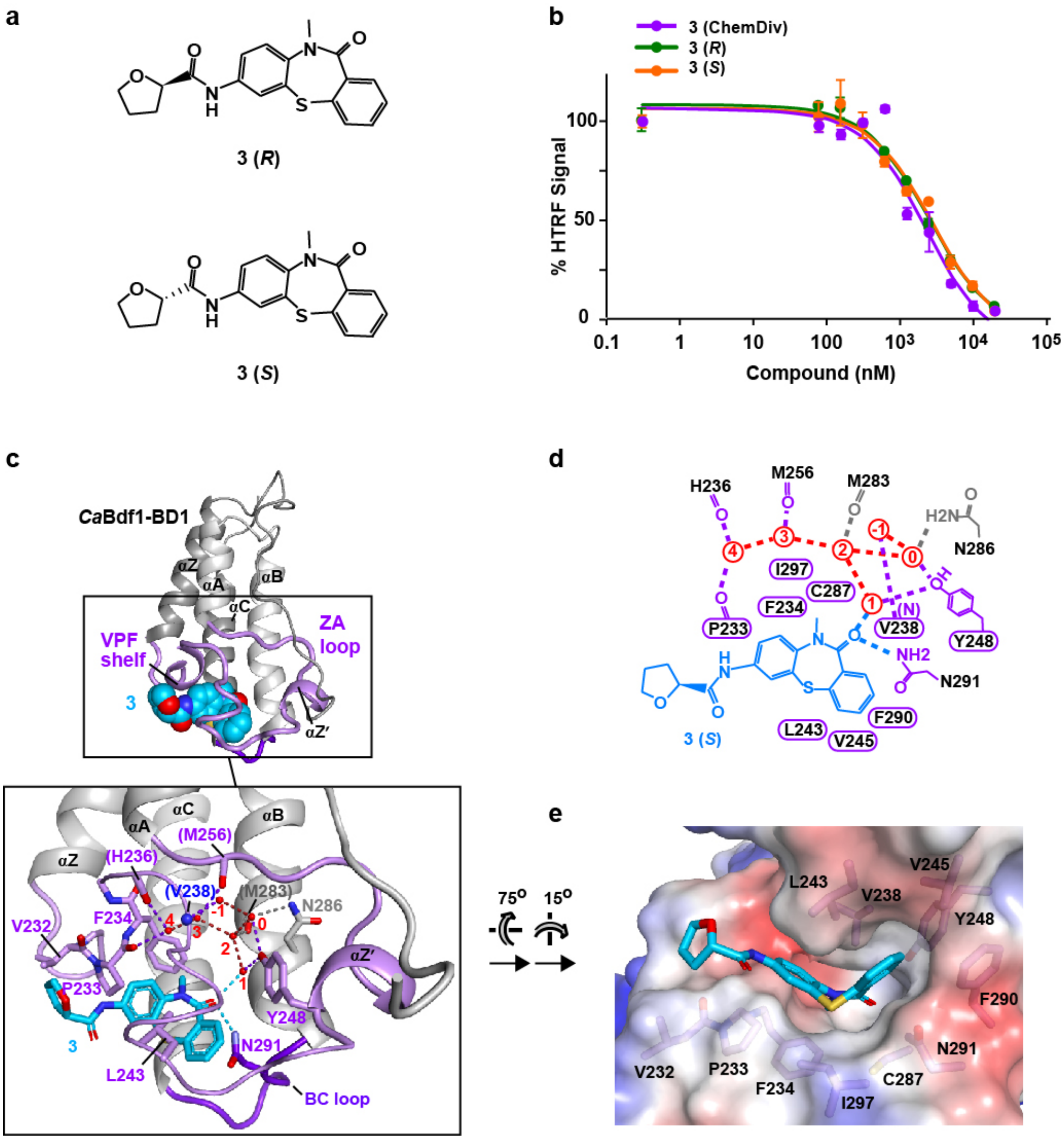

Supplementary Figure 9. Recognition of compound 3 by *Ca*Bdf1 BD1. (**a**) Chemical structure of *R* and *S* enantiomers of **3**. (**b**) HTRF assays showing that both enantiomers of **3** inhibit *Ca*Bdf1 BD1 with similar activity. The compound from ChemDiv used in the primary screen is a mix of *R* and *S* enantiomers. Data represent the mean and s.d. from three independent experiments. (**c**) Crystal structure of *Ca*Bdf1 BD1 bound to **3**(*S*). *Inset,* Details of the active site. Residues interacting with **3** through direct and water-mediated hydrogen bonds (dashed lines) are shown. The two hydrogen bonds to atoms of compound **3** are shown in cyan. Hydrogen bonds from water molecules to the ZA loop, to helix B or to other water molecules are in purple, gray, and red, respectively. Residues interacting through backbone atoms are labelled in parentheses. (**d**) Schematic summary of interactions. Hydrogen bonds are shown as dashed lines and coloured as in (c). (**e**) Electrostatic surface representation of *Ca*Bdf1 BD1 showing the region surrounding the bound inhibitor. The view is that of **Fig. 8d**.

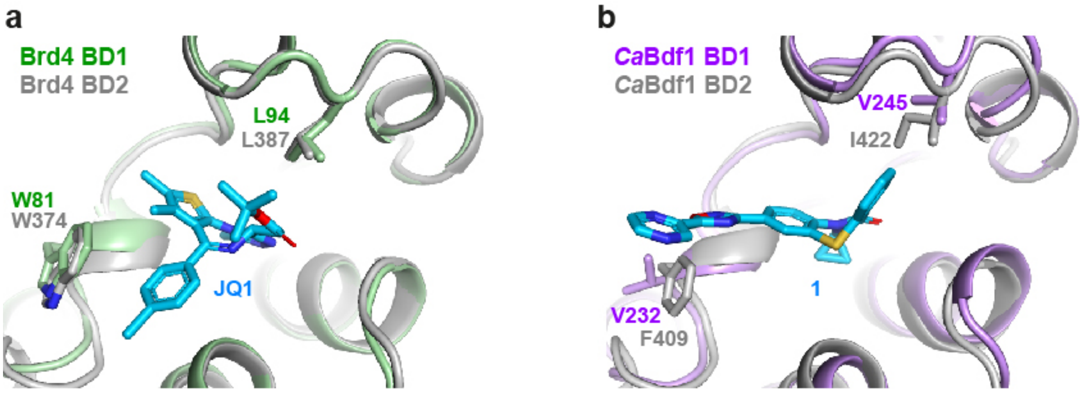

Supplementary Figure 10. Signature positions 1 and 3 in the BD1 and BD2 binding pockets. (**a**) Binding pocket of human Brd4 BD1 (green) and BD2 (green) showing conservation of the Trp and Leu residues at signature positions 1 and 3. (**b**) Binding pocket of *Ca*Bdf1 BD1 (violet) and BD2 (gray) showing divergence at signature positions 1 (Val232 and Phe409) and 3 (Val245 and Ile422).

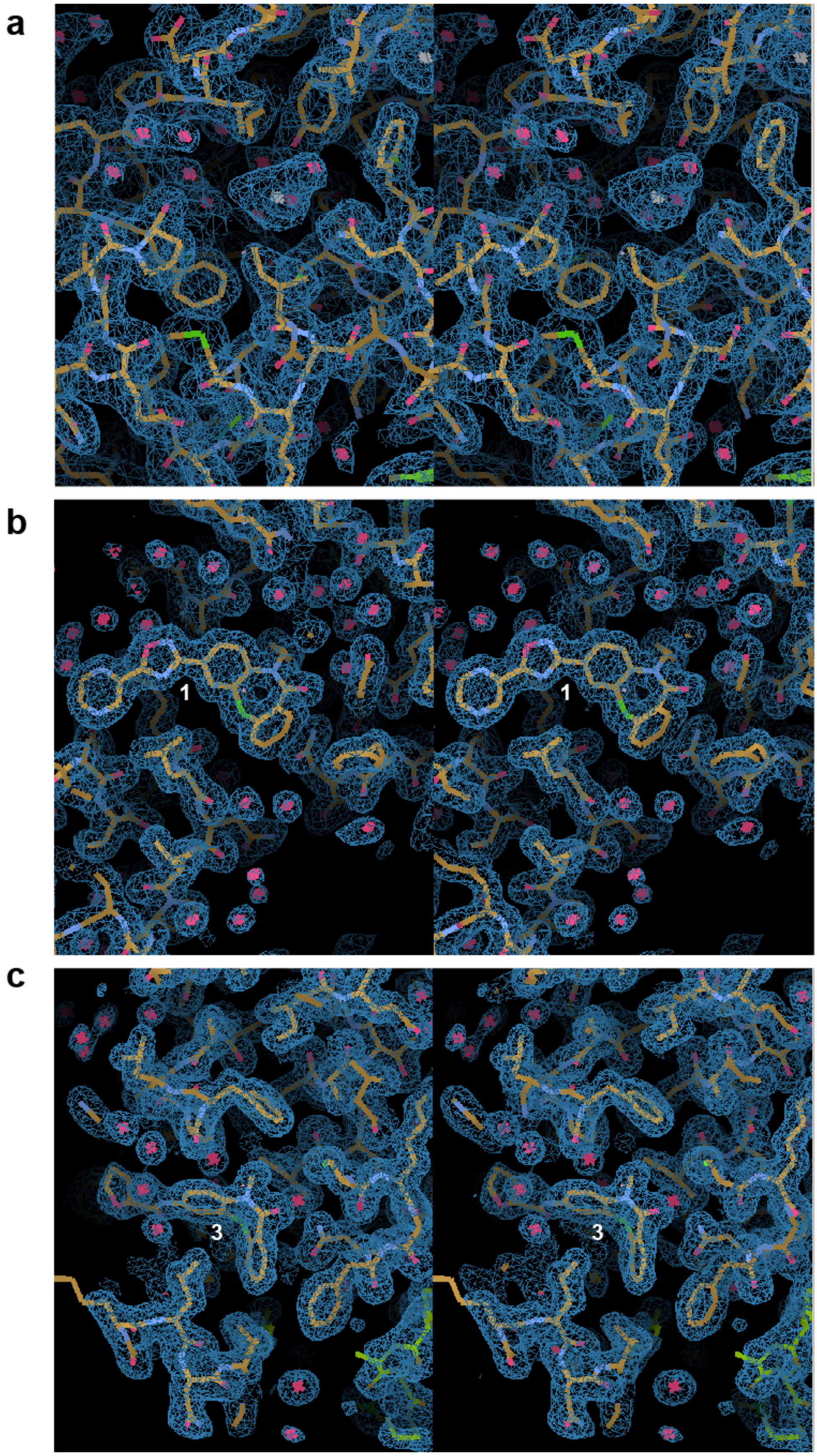

Supplementary Figure 11. Stereo images of final 2F_o-F_c density for crystal structures of *Ca*Bdf1 BD1. Density countoured at the 1σ level is shown for the ligand binding site (**a**) in the unbound state, (**b**) bound to compound **1** and (**c**) bound to compound **3**.

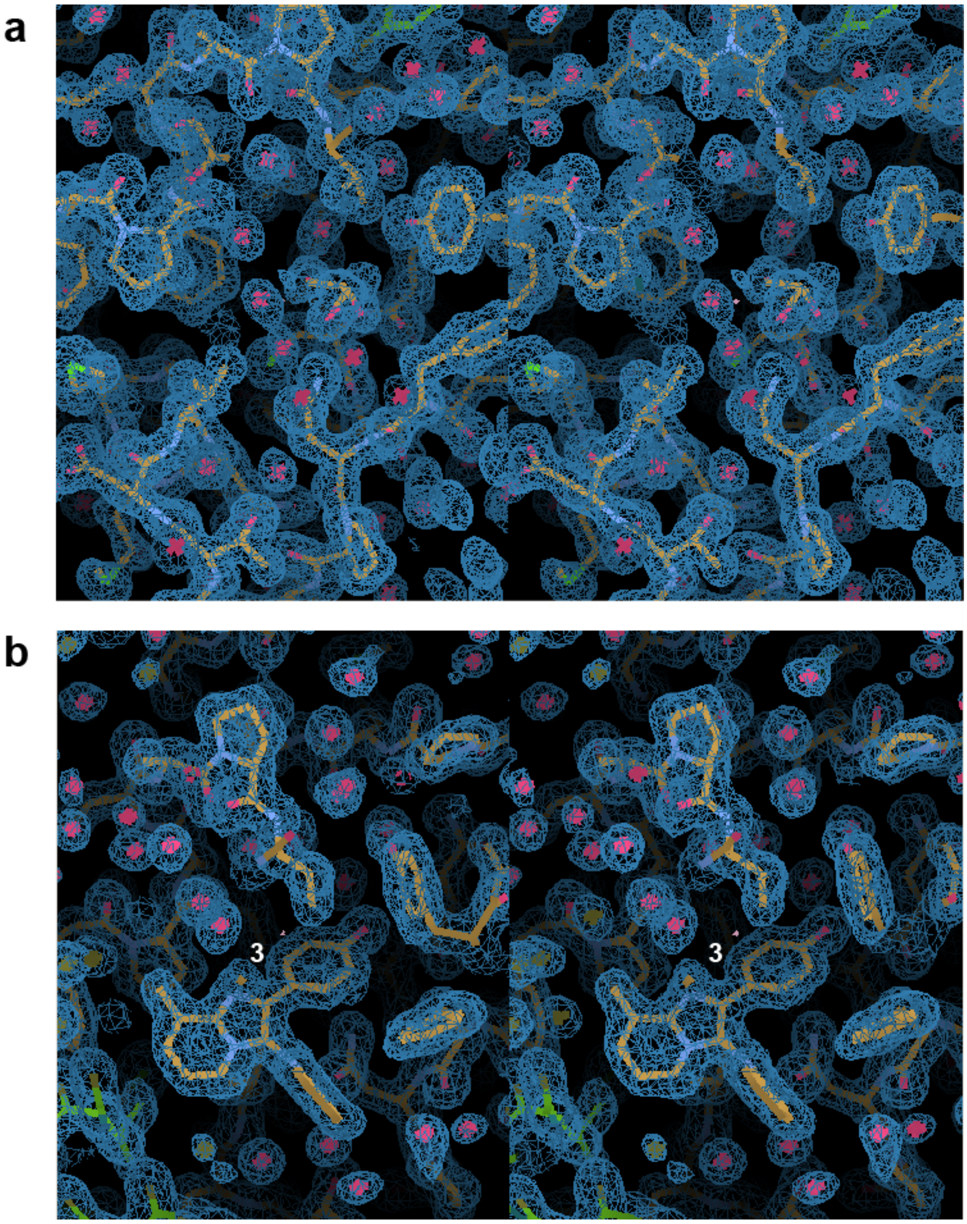

Supplementary Figure 12. Stereo images of final 2Fo-Fc density for crystal structures of *Ca*Bdf1 BD2. Density countoured at the 1σ level is shown for the ligand binding site (**a**) in the unbound state and (**b**) bound to compound **2**.

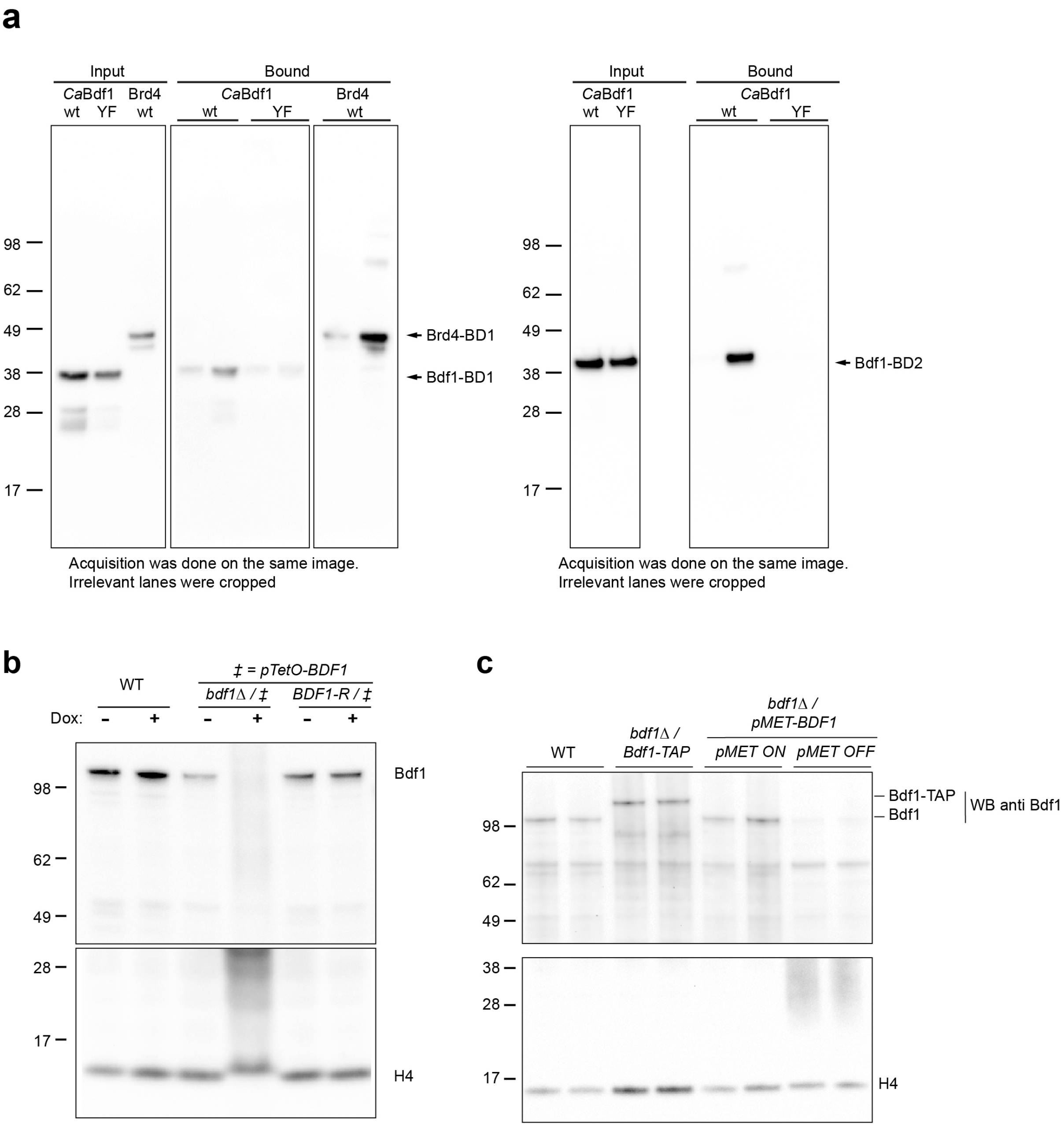

Supplementary Figure 13. Images of full Western blots. Blots correspond to (**a**) Fig. 1f (**b**) Fig. 2b and (**c**) Supplementary Fig. 1d**.**

Supplementary Table 1. ITC data for Brd4 and *Ca*Bdf1 BDs with JQ1 and compounds 1-3.

Inhibitor	Protein	[Inhibitor] (μM)	[Protein] (μM)	K_D (μM)	ΔH (kJ/mol)	N	$T\Delta S$ (kJ/mol)	ΔG (kJ/mol)
JQ1	Brd4 BD1	10	60	0.062 ± 0.016	-53.4 ± 0.5	1.00 ± 0.03	-12.28	-41.12
JQ1	*Ca*Bdf1 BD1	10	60	nd[1]	nd	nd	nd	nd
JQ1	*Ca*Bdf1 BD2	10	60	nd	nd	nd	nd	nd
**1**	Brd4 BD1	100	908	nd	nd	nd	nd	nd
**1**	*Ca*Bdf1 BD1	100	908	5.0 ± 2.5	-9.8 ± 2.6	0.91 ± 0.06	20.44	-30.24
**1**	*Ca*Bdf1 BD2	100	908	nd	nd	nd	nd	nd
**1**	SMARCA2 BD	100	908	nd	nd	nd	nd	nd
**1**	SMARCA4A BD	100	908	nd	nd	nd	nd	nd
**2**	*Ca*Bdf1 BD1	80	560	nd	nd	nd	nd	nd
**2**	*Ca*Bdf1 BD2	80	560	2.1 ± 0.2	-24 ± 4	1.21 ± 0.11	8.39	-32.39
**2**	SMARCA2 BD	80	560	nd	nd	nd	nd	nd
**2**	SMARCA4A BD	80	560	nd	nd	nd	nd	nd
**3**	Brd4 BD1	100	700	13.4 ± 1.7	-14.8 ± 1.9	0.92 ± 0.03	13.00	-27.80
**3**	*Ca*Bdf1 BD1	100	700	4.8 ± 0.2	-28.5 ± 0.5	1.05 ± 0.03	1.84	-30.34
**3**	*Ca*Bdf1 BD2	100	700	nd	nd	nd	nd	nd

[1]nd, not determined (weak binding).

Supplementary Table 2. Crystallographic data collection and refinement statistics.

Bromodomain:	***Ca*Bdf1 BD1 (unbound)**	***Ca*Bdf1 BD1 + compound 1**	***Ca*Bdf1 BD1 + compound 3**	***Ca*Bdf1 BD2 (unbound)**	***Ca*Bdf1 BD2 + compound 2**
**PDB ID**	**5N15**	**5N16**	**5N17**	**5N13**	**5N18**
**Data Collection**[1]					
ESRF beamline	ID23-2	ID30A-1	ID30A-1	ID29	ID30A-1
Wavelength (Å)	0.8726	0.9650	0.9660	0.9724	0.9660
Space group	$P2_12_12_1$	$P2_12_12_1$	$P4_12_12$	$P2_12_12_1$	P2
Unit cell dimensions	*a*=50.63 Å, *b*=67.57 Å, *c*=188.54 Å	*a*=75.44 Å, *b*=77.29 Å, *c*=102.59 Å	*a*=*b*=70.41 Å, *c*=124.17 Å	*a*=38.75 Å, *b*=45.20 Å, *c*=71.83 Å	*a*=46.49 Å, *b*=36.00 Å, *c*=64.50 Å, β=107.61
Molecules in asym. unit	4	4	2	1	2
Resolution range (Å)[2]	54.9 – 2.37 (2.45 – 2.37)	54.1 – 1.76 (1.82 – 1.76)	49.8 – 1.60 (1.66 – 1.60)	45.2 – 1.20 (1.24 – 1.20)	44.3 – 1.45 (1.50 – 1.45)
No. of measured reflections	132,375 (13,158)	266,479 (25,764)	313,833 (26,686)	229,797 (9523)	84,954 (8522)
No. of unique reflections	27,138 (2644)	59,921 (5873)	41,446 (4131)	39,415 (3330)	34,661 (3549)
Multiplicity	4.9 (5.0)	4.4 (4.4)	7.6 (6.5)	5.8 (2.9)	2.45 (2.40)
Completeness (%)	99.8 (99.9)	99.5 (99.3)	98.3 (99.6)	97.9 (83.8)	95.2 (97.3)
Mean I/sigma(I)	14.5 (2.3)	12.1 (2.3)	16.1 (2.5)	17.0 (3.0)	12.1 (2.6)
R_{merge}	0.088 (0.789)	0.070 (0.626)	0.067 (0.689)	0.050 (0.263)	0.047 (0.442)
R_{meas}	0.098 (0.881)	0.080 (0.708)	0.072 (0.748)	0.055 (0.321)	0.059 (0.549)
R_{pim}	0.044 (0.388)	0.037 (0.324)	0.025 (0.285)	0.022 (0.180)	0.035 (0.321)
$CC_{1/2}$	0.998 (0.761)	0.998 (0.759)	0.998 (0.812)	0.999 (0.918)	0.998 (0.805)
**Refinement**					
Resolution	48.9 – 2.37	42.7 – 1.76	49.8 – 1.60	38.3 – 1.20	44.3 – 1.45
No. reflections (total/R_{free})	25,719 / 1369	56,817 / 3041	39,332 / 2092	37,375 / 1980	32,851 / 1790
R_{work}/R_{free}	0.2027 / 0.2464	0.1785 / 0.2148	0.2131 / 0.2352	0.1426 / 0.1560	0.1593 / 0.1978
Number of atoms/Mean *B*-factor ($Å^2$)					
Protein	4193 / 49.5	4110 / 26.2	2062 / 35.8	877 / 13.4	1736 / 17.3
Water	126 / 42.0	648 / 36.7	259 / 39.3	194 / 30.8	273 / 30.4
Inhibitor	0 / --	120 / 19.5	86 / 38.6	0 / --	25 / 15.9
Other	44 / 100.6	11 / 33.8	10 / 70.3	12 / 18.2	6 / 67.8
R.m.s. deviations:					
Bond distances (Å)	0.002	0.006	0.0038	0.004	0.004
Bond angles (^{o})	0.454	0.979	0.857	0.763	0.801
Ramachandran analysis (%)					
Favored/ outliers	98.1 / 0.0	99.8 / 0.0	97.3 / 0.0	98.1 / 0.0	100.0 / 0.0
Molprobity analysis					
Clash Score / Overall score	2.34 / 1.03	2.96 / 1.09	3.71 / 1.30	1.14 / 0.82	2.60 / 1.05

[1] A single crystal was used for each structure.

[2] Numbers in parentheses refer to the outer resolution shell.

Supplementary Table 3. RMSD values between human BET and *Ca*Bdf1 BD structures.

	RMSD (Å) with *Ca*Bdf1	
	**Entire BD (100 Cα atoms)**	**Binding pocket (46 Cα atoms)**
**BD1**		
Brd2	1.79	0.53
Brd3	1.41	0.39
Brd4	1.36	0.56
Brdt	1.28	0.53
**Mean**	**1.46 ± 0.23**	**0.50 ± 0.08**
**BD2**		
Brd2	1.12	1.36
Brd3	1.10	1.38
Brd4	1.19	1.50
Brdt	1.09	1.39
**Mean**	**1.13 ± 0.05**	**1.41 ± 0.06**

Supplementary Table 4. Primers used in this study.

Construction	Number	Sequence
His Bdf1-BD1 (193-327)	prJG339	GGCGCCATGGGAGCTCCCAAACCACCACAAGAACCAGATAT
	prJG340	CGCCGAATTCTCATGCAACGTTAGTTCCAGCTGGCAACTCTT
His Bdf1-BD2 (386-491)	prJG450	CGCGGATCCCCGGCAGCAGAATTAAGATTTTG
	prJG451	CGCGCTCGAGTCAGTTGGCCCATTTCTTGTCA
GST Bdf1-BD1 (193-327)	prJG440	CGCGGATCCCCGGCTCCCAAACCACCACAAGAA
	prJG441	CGCGCTCGAGTCATGCAACGTTAGTTCCAGCTGGCA
GST Bdf1-BD2 (361-501)	prJG457	CGCGGATCCCCGAGACCAAAGAGAACTATCCATCCAC
	prJG458	CGCGGATCCCCGCGAGATTCAGTGGCTGCA
Bdf1-BD1 Y248F	prJG572	GGACACTGTCAAATTAAATGTCCCATTCGCTTACAATTACATTCCAAGACC
	prJG573	GGTCTTGGAATGTAATTGTAAGCGAATGGGACATTTAATTTGACAGTGTCC
Bdf1-BD1 Y425F	prJG574	GGATACAGTAGCTTTGAACATACCTAACGCTAATGAAATAGTGAAGCAACCAATGG
	prJG575	CCATTGGTTGCTTCACTATTTCATTAGCGTTAGGTATGTTCAAAGCTACTGTATCC

Supplementary Table 5. Plasmids used in this study.

Name	Number	Parent	Description	Cassette to transform
5'BDF1-HIS1-3'BDF1	pJG197		pCR2.1 TOPO containing HIS1 marker flanked by upstream and downstream BDF1 sequence	PCR
bdf1-LEU2	pJG214		pCR2.1 TOPO containing BDF1 ORF fused to LEU2 marker and *BDF1* downstream region	Digestion with ApaI
bdf1-bd1Δ-LEU2	pJG224		Identical to pJG214 with a bdf1-bd1Δ ORF	" " "
bdf1-bd2Δ-LEU2	pJG225		Identical to pJG214 with a bdf1-bd2Δ ORF	" " "
bdf1-bd1Δ-bd2Δ-LEU2	pJG226		Identical to pJG214 with a bdf1-bd1Δ-bd2Δ ORF	" " "
bdf1-bd1Y248F-LEU2	pJG215	pJG214	Identical to pJG214 with a bdf1-bd1Y248F ORF	" " "
bdf1-bd2Y425F-LEU2	pJG216	pJG214	Identical to pJG214 with a bdf1-bd2Y425F ORF	" " "
bdf1-bd1Y248F-bd2Y425F-LEU2	pJG217	pJG215	Identical to pJG214 with a bdf1-bd1Y248F-bd2Y425F sequence	" " "
ARG4-pTetO-bdf1	pJG254		pCR2.1 TOPO containing ARG4 marker, tTA-TetR-VP16, Tet operator and flanking regions for integration in *BDF1* promoter	Digestion with XhoI
His Bdf1-BD1 (193-327)	pJG177	pETM11	Production of Bdf1-BD1	
His Bdf1-BD2 (386-491)	pJG182	pETM11	Production of Bdf1-BD2	
His Brd4-BD1 (22-204)			Production of Brd4-BD1	
GST Bdf1-BD1 (193-327)	pJG187	pGEX4t1	Production of Bdf1-BD1	
GST Bdf1-BD1 (193-327) Y248F		pGEX4t1	Production of Bdf1-BD1 Y248F	
GST Bdf1-BD2 (361-501)	pJG193	pGEX4t1	Production of Bdf1-BD2	
GST Bdf1-BD2 (361-501) Y425F		pGEX4t1	Production of Bdf1-BD2 Y425F	

Supplementary Table 6. *Candida albicans* strains used in this study.

Name[1]	Number	Parent	Genotype
SN152	SN152	SC5314	*ura3/::imm434::URA3/ura3::imm434 iro1::IRO1/iro1::imm434 his1::hisG/his1::hisG leu2/leu2 arg4/arg4*
bdf1Δ / pTetO-BDF1	yCaJG105	SN152	*SN152 + bdf1::pTetO-BDF1-ARG4/bdf1Δ::HIS1*
BDF1-R / pTetO-BDF1	yCaJG108	yCaJG105	*SN152 + bdf1::pTetO-BDF1-ARG4/bdf1::BDF1-R-LEU2*
bdf1-bd1Δ / pTetO-BDF1	yCaJG120	"	*SN152 + bdf1::pTetO-BDF1-ARG4/bdf1::bdf1-bd1D-LEU2*
bdf1-bd2Δ / pTetO-BDF1	yCaJG123	"	*SN152 + bdf1::pTetO-BDF1-ARG4/bdf1::bdf1-bd2D-LEU2*
bdf1-bd1Δ-bd2Δ/ pTetO-BDF1	yCaJG127	"	*SN152 + bdf1::pTetO-BDF1-ARG4/bdf1::bdf1-bd1D-bd2D-LEU2*
bdf1-bd1Y248F/ pTetO-BDF1	yCaJG111	"	*SN152 + bdf1::pTetO-BDF1-ARG4/bdf1::bdf1-bd1Y248F-LEU2*
bdf1-Bd2Y425F/ pTetO-BDF1	yCaJG114	"	*SN152 + bdf1::pTetO-BDF1-ARG4/bdf1::bdf1-bd2Y425F-LEU2*
bdf1-bd1Y248F-bd2Y425F/ pTetO-BDF1	yCaJG117	"	*SN152 + bdf1::pTetO-BDF1-ARG4/bdf1::bdf1-bd1Y248F-bd2Y425F-LEU2*
bdf1Δ/BDF1-TAP	yCaJG49	SN152	*SN152 + BDF1::BDF1-TAP-LEU2/bdf1Δ::HIS1*
bdf1Δ / pMET-BDF1	yCaJG31	SN152	*SN152 + bdf1::pMET-BDF1-ARG4/bdf1Δ::HIS1*
BDF1-R / pMET-BDF1	yCaJG37	yCaJG31	*SN152 + bdf1::pMET-BDF1-ARG4/bdf1::BDF1-R-LEU2*
bdf1-bd1Δ / pMET-BDF1	yCaJG81	"	*SN152 + bdf1::pMET-BDF1-ARG4/bdf1::bdf1-bd1D-LEU2*
bdf1-bd2Δ / pMET-BDF1	yCaJG84	"	*SN152 + bdf1::pMET-BDF1-ARG4/bdf1::bdf1-bd2D-LEU2*
bdf1-bd1Δ-bd2Δ/ pMET-BDF1	yCaJG87	"	*SN152 + bdf1::pMET-BDF1-ARG4/bdf1::bdf1-bd1D-bd2D-LEU2*
bdf1-bd1Y248F/ pMET-BDF1	yCaJG40	"	*SN152 + bdf1::pMET-BDF1-ARG4/bdf1::bdf1-bd1Y248F-LEU2*
bdf1-Bd2Y425F/ pMET-BDF1	yCaJG43	"	*SN152 + bdf1::pMET-BDF1-ARG4/bdf1::bdf1-bd2Y425F-LEU2*
bdf1-bd1Y248F-bd2Y425F/ pMET-BDF1	yCaJG46	"	*SN152 + bdf1::pMET-BDF1-ARG4/bdf1::bdf1-bd1Y248F-bd2Y425F-LEU2*

[1] Strain SN152 is from ref.[5]. All other strains are from this study

SUPPLEMENTARY METHODS

Synthesis of inhibitors

1-chloro-2,4-dinitrobenzene, 2-mercaptobenzoic acid, (*R*)-tetrahydrofuran-2-carboxylic acid and (*S*)-tetrahydrofuran-2-carboxylic acid were purchased from Sigma-Aldrich. 2-bromo-1-(4-hydroxyphenyl)ethan-1-one, 2-bromo-1-(4-fluorophenyl)ethan-1-one and 3- methylpyridin-2-amine were purchased from TCI America. *p*-toluidine was purchased from Alfa Aesar. 2-bromo-1-phenylethan-1-one was purchased from Oxchem Corp. All other reagents were purchased from commercial sources and used as obtained. Compounds were synthesized as described below. ^{1}H, ^{19}F NMR spectra were obtained on Varian 400-MR and VNMRS-600 NMR Spectrometers. The chemical shifts are relative to external hexafluorobenzene, C_6F_6 (δ - 164.9, ^{19}F NMR). Multiplicities are quoted as singlet (s), doublet (d), triplet (t) unresolved multiplet (m), doublet of doublets (dd), doublet of doublet of doublets (ddd), doublet of triplets (dt) or broad signal (br). All chemical shifts are given on the δ-scale in parts per million (ppm) relative to internal CD_2HOD (δ 3.34, ^{1}H NMR), CHCl3 (δ 7.26, ^{1}H NMR). ^{1}H, ^{19}F coupling constants (*J* value) are given in Hz. The concentration of the NMR samples was in the range of 4-6 mg/mL. Normal phase chromatography was performed using ISCO Combiflash Lumen+. UV and ELS detectors were used. Mass Spectrometry (MS) was performed on a Finnigan LCQ Deca XP Max mass spectrometer equipped with an ESI source in the negative ion mode. The IUPAC names of compounds were assigned using MarvinSketch. All the NMRs were processed and interpreted using MestReNova 9.0.0.

Synthesis of Enantiomers of 3

The individual enantiomers of **3** were synthesized by a modification of the method previously reported by Smirnov *et al.*[6].

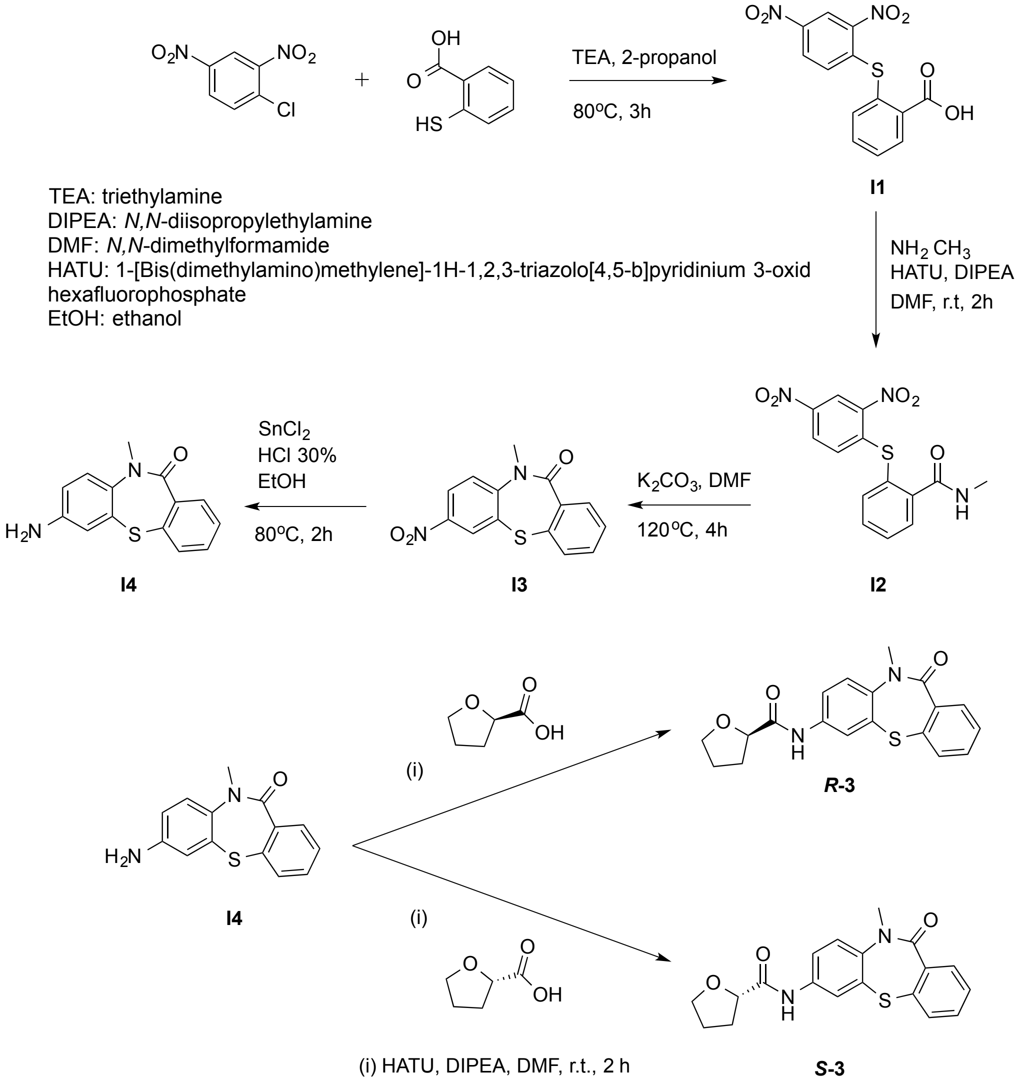

0.5 g (2.5 mmol, 1 equiv.) 1-chloro-2,4-dinitrobenzene and 0.38 g (2.5 mmol, 1 equiv.) 2-mercaptobenzoic acid were added to a mixture of 0.68 mL TEA and 3.7 mL 2-propanol. The mixture was heated to 80°C with vigorous stirring for 3 hr. The product was extracted with EtOAc (5 mL x 3). The solvent was then removed under vacuum to get intermediate **I1** (2-((2,4-dinitrophenyl)thio)benzoic acid, 90% yield) for the next step. 0.5 g (1.6 mmol, 1 equiv.) 2-((2,4-dinitrophenyl)thio)benzoic acid **I1** was added to 8.25 mL DMF. 0.713 g (1.2 equiv.) HATU and 0.404 g (2 equiv.) DIPEA were then added to the solution. 0.0586 g (1.1 equiv.) NH_2CH_3 was also added to the mixture. The reaction mixture was stirred vigorously at RT for 2 hr. Intermediate **I2** was extracted with EtOAc (5 mL x 3) and dried under vacuum. The resulting product was further purified by silica gel column chromatography (Hexane/EtOac 0-100%) (70% yield). 0.26 g (1 equiv.) of the product and 0.04 g (2 equiv.) K_2CO_3 were then placed in a 10 mL round bottom flask with 2 mL DMF. The mixture was kept at 120°C (oil bath) with vigorous stirring for 4 hr. After solvent removal in vacuum the resulting compound **I3** was washed with DI water and EtOH and dried to get the product (49% yield). 95 mg (1 equiv.) of **I3** was dissolved in EtOAc (3 mL) and mixture of 1mL EtOH and 300μL (7 equiv.) 30% HCl was then added to the solution. Finally, 0.262 g (3.5 equiv.) $SnCl_2$ was added to the reaction mixture. The mixture was heated at 80°C with vigorous stirring for 2 hr. After reaction completion (monitored by TLC) the pH was adjusted to 11 with NaOH. Formed precipitate **I4** was washed 3 times with water and dried under vacuum. Yield 42%. Then 32 mg (1equiv.) of **I4** and 15.44 mg (1.1 equiv.) of (*R*)- tetrahydrofuran-2-carboxylic acid (or (*S*)-tetrahydrofuran-2-carboxylic acid) were dissolved in 1 mL DMF. 54.75 mg (1.2 equiv.) HATU and 31.02 mg (2 equiv.) DIPEA were then added to the solution. The reaction was vigorously stirred at RT for 2 hr. Product was extracted by EtOAc (5 mL x 3) and dried under vacuum. The resulting product was further purified by silica gel column chromatography (Hexane/EtOac 0-100%) and the final compound [**3**(*R*) or *S*-**3**(*S*)] was obtained with 42% yield (18.4 mg).

(*R*)-N-(10-methyl-11-oxo-10,11-dihydrodibenzo[b,f][1,4]thiazepin-7- yl)tetrahydrofuran-2-carboxamide, **the R-enantiomer of 3**, structure:

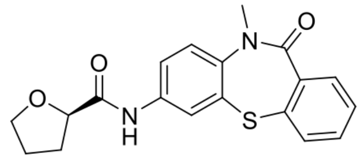

^{1}H NMR (600 MHz, $CDCl_3$): δ 8.47 (s, 1H), 7.81 (dd, *J* = 46.3, 2.5 Hz, 1H), 7.75 – 7.69 (m, 1H), 7.59 (ddd, *J* = 49.7, 8.7, 2.5 Hz, 1H), 7.42 (dt, *J* = 7.3, 2.1 Hz, 1H), 7.34 – 7.27 (m, 2H), 7.25 (d, *J* = 2.7 Hz, 1H), 4.43 (dd, *J* = 8.4, 5.9 Hz, 1H), 4.00 (t, *J* = 7.2 Hz, 1H), 3.94 (q, *J* = 7.3 Hz, 1H), 3.58 (s, 3H), 2.35 (dd, *J* = 13.4, 7.3 Hz, 1H), 2.13 (tt, *J* = 8.2, 2.9 Hz, 1H), 2.00 – 1.83 (m, 2H); MS (m/z): $[M]^+$ calcd. for $C_{19}H_{18}N_2O_3S$, 354.10; found: 355.3 $[M+H]^+$, 709.0 $[2M+H]^+$, 731.1 $[2M+Na]^+$.

(*S*)-N-(10-methyl-11-oxo-10,11-dihydrodibenzo[b,f][1,4]thiazepin-7- yl)tetrahydrofuran-2-carboxamide, **the S-enantiomer of 3**, structure:

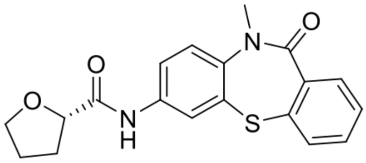

^{1}H NMR (400 MHz, $CDCl_3$); δ 8.51 (s, 1H), 7.83 (dd, *J* = 30.2, 2.5 Hz, 1H), 7.77 – 7.68 (m, 1H), 7.60 (ddd, *J* = 32.9, 8.7, 2.5 Hz, 1H), 7.47 – 7.40 (m, 1H), 7.34 – 7.25 (m, 3H), 4.44 (dd, *J* = 8.4, 5.9 Hz, 1H), 4.02 (qd, *J* = 7.1, 1.1 Hz, 1H), 3.98 – 3.89 (m, 1H), 3.59 (s, 3H), 2.85 (s, 2H), 2.42 – 2.30 (m, 1H), 2.20 – 2.08 (m, 1H), 1.94 (dtt, *J* = 19.6, 12.6, 6.7 Hz, 2H); ^{13}C NMR (101 MHz, $CDCl_3$): δ 171.52, 168.98, 140.73, 137.78, 134.98, 131.66, 131.05, 130.89, 128.57, 125.19, 123.56, 123.43, 120.68, 78.48, 77.04, 69.71, 30.17, 25.56; MS (m/z): $[M]^+$ calcd. for $C_{19}H_{18}N_2O_3S$, 354.10; found, 355.6 $[M+H]^+$, 377.4 $[M+Na]^+$, 730.9 $[2M+Na]^+$.

Synthesis of 2, 2a and 2b

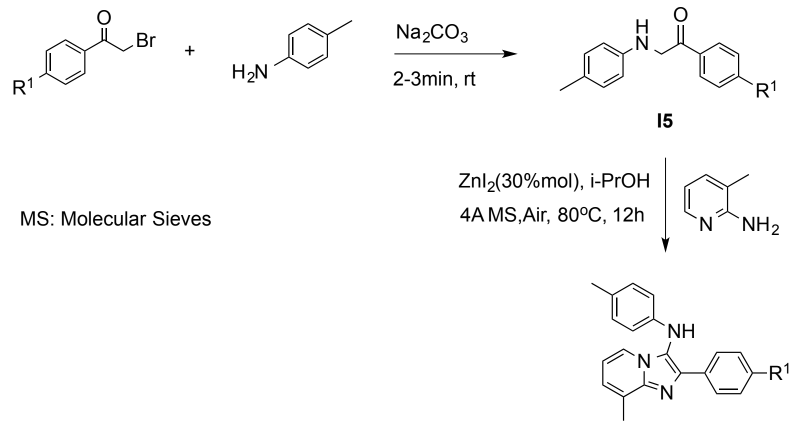

R[1]= OH (**2**), H (**2a**), F (**2b**)

2 mmol (1 equiv) of corresponding 2-bromoacetophenone and 0.235 g (2.2 mmol, 1.1 equiv.) *p*-toluidine were added to a mortar. 0.1 g Na_2CO_3 was added and the mixture was grinded for 2 min at RT until it became yellow[7]. The crude material was washed 3 times with water and dried under the vacuum giving solid material **I5** with yields 80-90%. 1 mmol (1 equiv.) of **I5** was then dissolved in 6 mL isopropyl alcohol and 0.108 g 3-methylpyridin-2- amine (1 mmol, 1 equiv.) was added dropwise to the solution. Finally, 0.1 g ZnI_2 and 0.5 g 4 Å molecular sieves were added. The mixture was heated at 80°C with vigorous stirring for 12 hr[8]. After reaction completion (monitored by TLC), the product was extracted with EtOAc (5 mL x 3) and washed with DI water (5 mL x 3). The organic layer was dried over Na_2SO_4 and the solvent was removed under vacuum. The resulting product was further purified by silica gel column chromatography (Hexane/EtOAc 0-90%), giving target compounds **2**, **2a** or **2b** with yields 18-24%.

4-(8-methyl-3-(*p*-tolylamino)imidazo[1,2-a]pyridin-2-yl)phenol, **2**, structure:

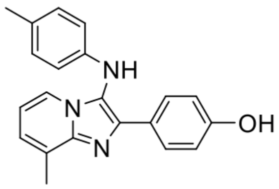

^{1}H NMR (400 MHz, CD_3OD): δ 7.79 (d, *J* = 8.5 Hz, 3H), 7.11 (d, *J* = 6.8 Hz, 1H), 6.94 (d, *J* = 8.4 Hz, 2H), 6.81 – 6.75 (m, 3H), 6.42 (d, *J* = 8.4 Hz, 2H), 2.62 (s, 3H), 2.20 (s, 3H); MS (m/z): $[M]^+$ calcd. for $C_{21}H_{19}N_3O$, 329.15; found, 330.4 $[M+H]^+$.

8-methyl-2-phenyl-N-(p-tolyl)imidazo[1,2-a]pyridin-3-amine, **2a**, structure:

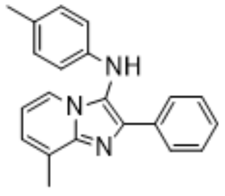

^{1}H NMR (400 MHz, CD_3OD): δ 7.97 – 7.91 (m, 2H), 7.88 (d, *J* = 6.7 Hz, 1H), 7.41 – 7.35 (m, 2H), 7.34 – 7.30 (m, 1H), 7.23 (dt, *J* = 6.9, 1.1 Hz, 1H), 6.96 (d, *J* = 8.0 Hz, 2H), 6.89 (t, *J* = 6.8 Hz, 1H), 6.48 – 6.36 (m, 2H), 2.65 (s, 3H), 2.20 (s, 3H); ^{13}C NMR (101 MHz, $CDCl_3$) δ 142.83, 142.38, 130.26, 129.05, 128.53, 127.68, 127.54, 127.23, 123.84, 120.72, 118.81, 113.45, 112.25, 20.44, 16.66.MS (m/z): $[M]^+$ calcd. for $C_{21}H_{19}N_3$, 313.16; found, 314.5 $[M+H]^+$.

2-(4-fluorophenyl)-8-methyl-N-(p-tolyl)imidazo[1,2-a]pyridin-3-amine, **2b**, structure:

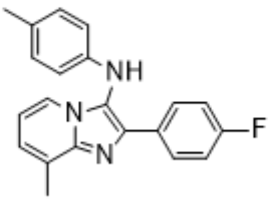

^{1}H NMR (400 MHz, $CDCl_3$): δ 8.01 (dd, J = 8.8, 5.5 Hz, 2H), 7.69 (d, J = 6.7 Hz, 1H), 7.06 – 6.95 (m, 5H), 6.69 (t, J = 6.8 Hz, 1H), 6.49 (d, J = 8.4 Hz, 2H), 2.68 (s, 3H), 2.25 (s, 3H); ^{13}C NMR (101 MHz, $CDCl_3$) δ 162.39 (d, $^1J_{CF}$ = 247.3 Hz), 142.35, 142.14, 137.22, 130.28, 129.09, 128.88 (d, $^3J_{CF}$ = 8.0 Hz), 127.14, 124.63, 120.78, (nr), 118.63, 115.32 (d, $^2J_{CF}$ = 22.9 Hz), 113.35, 112.57, 20.43, 16.74. ^{19}F NMR (564 MHz, $CDCl_3$): δ -105.23 , -106.62 , -114.35; MS (m/z): $[M]^+$ calcd. for $C_{21}H_{18}FN_3$, 331.15; found, 332.3 $[M+H]^+$.

Supplementary References

1 Filippakopoulos, P. *et al.* Histone recognition and large-scale structural analysis of the human bromodomain family. *Cell* **149**, 214-231 (2012).

2 Filippakopoulos, P. *et al.* Selective inhibition of BET bromodomains. *Nature* **468**, 1067-1073 (2010).

3 Flynn, E. M. *et al.* A Subset of Human Bromodomains Recognizes Butyryllysine and Crotonyllysine Histone Peptide Modifications. *Structure* **23**, 1801-1814 (2015).

4 Winter, G. E. *et al.* DRUG DEVELOPMENT. Phthalimide conjugation as a strategy for in vivo target protein degradation. *Science* **348**, 1376-1381 (2015).

5 Noble, S. M. & Johnson, A. D. Strains and strategies for large-scale gene deletion studies of the diploid human fungal pathogen Candida albicans. *Eukaryot Cell* **4**, 298-309 (2005).

6 Smirnov, A. V., Kalandadze, L. S., Sakharov, V. N., Dorogov, M. V. & Ivachtchenko, A. V. Denitrocyclization in synthesis of dibenzo[b,f][1,4]thiazepin-11(10h)-ones and their derivatives. *J Heterocycl Chem* **44**, 1247-1251 (2007).

7 Gupta, G. K., Saini, V., Khare, R. & Kumar, V. 1,4-Diaryl-2-mercaptoimidazoles derivatives as a novel class of antimicrobial agents: design, synthesis, and computational studies. *Med Chem Res* **23**, 4209-4220 (2014).

8 Han, X., Ma, C. W., Wu, Z. Y. & Huang, G. S. Zinc Iodide Catalyzed Synthesis of 3-Aminoimidazo[1,2-a]pyridines from 2-Aminopyridines and α-Amino Carbonyl Compounds. *Synthesis* **48**, 351-356 (2016).
